# An unusual complex suicide involving a chainsaw and a hanging: a case report

**DOI:** 10.1186/s13256-022-03344-4

**Published:** 2022-03-28

**Authors:** Barbara Gualco, Amalia Angelino, Regina Rensi, Gloria Manetti, Martina Focardi

**Affiliations:** Division of Forensic Legal Medicine, Department of Health Sciences, University of Florence, Largo Brambilla, 3, 50134 Florence, Italy

**Keywords:** Hanging, Complex suicide, Chainsaw, Differential diagnosis

## Abstract

**Background:**

Suicides or suicidal attempts with power tools such as band or circular saws are rarely encountered in forensic medicine practice; in the forensic literature, only a few cases have been reported. We present the case of a “combined suicide” (self-injurious actions using different methods and involving various bodily sites) carried out with uncommon deadly methods: chainsaw and hanging.

**Case presentation:**

A 58-year-old Caucasian man was found dead by his wife, hanging from the basement ceiling of the cellar in which he lived. During the investigation of the crime scene, external examination revealed a wide incised wound in the umbilical area showing the muscular and adipose tissues below. On inspection of the garden around the courtyard, the police found a chainsaw with blood spatter on both the blade and the handle. The blade appeared to be compatible with the abdominal injuries.

**Conclusions:**

Since the corpse presented this incised wound, it was crucial to establish the manner and the causes of death, as well as to exclude any third-party involvement. In this case, the presence of multiple injuries, potentially lethal, required a differential diagnosis between suicide or homicide. A detailed post-mortem examination, in association with a precise study of the circumstantial data, led the authors to assume that they were dealing with a “complex suicide.”

## Background

The presented case is a “complex suicide” (self-injurious actions using different methods and involving various body parts) carried out with uncommon lethal methods: chainsaw and hanging. Suicides or suicidal attempts with power tools such as band or circular saws are rare events, with only a few cases reported in the forensic literature [1–12]. The injuries are almost exclusively inflicted on the head/neck region, causing death through central dysregulation, exsanguination, air embolism, or blood aspiration. Generally, suicidal self-infliction through such power tools is associated with psychiatric disease and alcohol/drug abuse; most of the decedents are male [13]. Suicides by chainsaws or circular saws are regarded as extreme exceptions [1, 4, 5, 7, 9–11].

The authors report the case of a 58-year-old Caucasian man who was found dead, hanged in the basement of the cellar with an extensive laceration in the umbilical area.

A rigorous analysis of the circumstantial elements, the investigation of the death scene, and, mostly, the autopsy findings seemed to fit with a case of suicide.

The purpose of the manuscript is to contribute to the literature regarding the complexity of complex suicide.

The ethics committee of the Department of Health Sciences (University of Florence, Italy) approved the publication of this case report.

It was not possible to request consent from the wife (the closest relative) because she cannot be found. In any case, Italian legislation does not require informed consent for the publication of case reports. The case was presented anonymously. It was written according to the rules of the Oviedo Convention.

## Case presentation

A 58-year-old Caucasian man was found dead by his wife, hanging from the ceiling in the basement of the cellar in which he used to live. The wife found the man 3 hours after death.

From the anamnestic data collected, the man had been suffering from severe depression for many years. He had retreated into seclusion for several years and moved to the cellar for no apparent reason many years before. The man was taking an antidepressant drug belonging to the class of selective serotonin reuptake inhibitors (SSRIs): Prozac (three tablets per day, 60 mg). His everyday routine used to consist of taking care of the kitchen garden in the morning and keeping himself busy with carpentry work in the afternoon.

There are previous records of suicide attempts, 2 years earlier, by consuming alcohol together with the antidepressant drugs he had been regularly taking for his therapy.

The first inspection of the death scene led to the belief that it was a suicide, but since a large amount of blood and some lacerations were noted on the sweater, a forensic pathologist was required. Preliminary examination of the body, once the sweater had been lifted, revealed a wide incised wound, with irregular edges and of irregular shape, in the umbilical area, showing the muscular and adipose tissues below.

In consideration of these injuries, a careful inspection of the scene was carried out, but no further blood traces were found, except for a small pool of blood in the area below the body. On inspection of the surrounding area, the police found a chainsaw with blood spatter on both the blade and the handle in the garden beside the courtyard. Some textile fragments of the same color as the sweater were also discovered near the chainsaw in the garden.

**Fig. 1:**
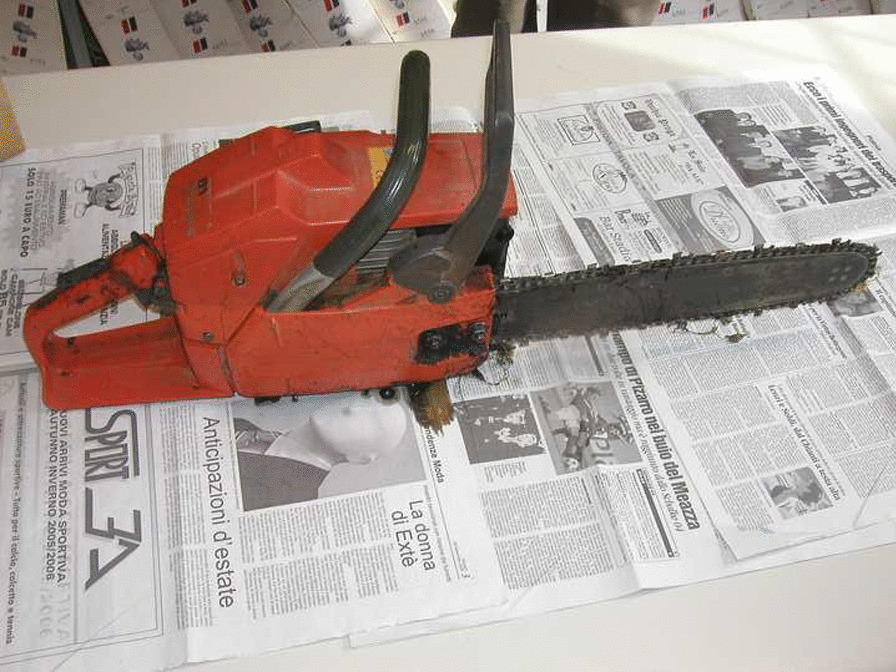
The chainsaw used in the presented case

**Fig. 2:**
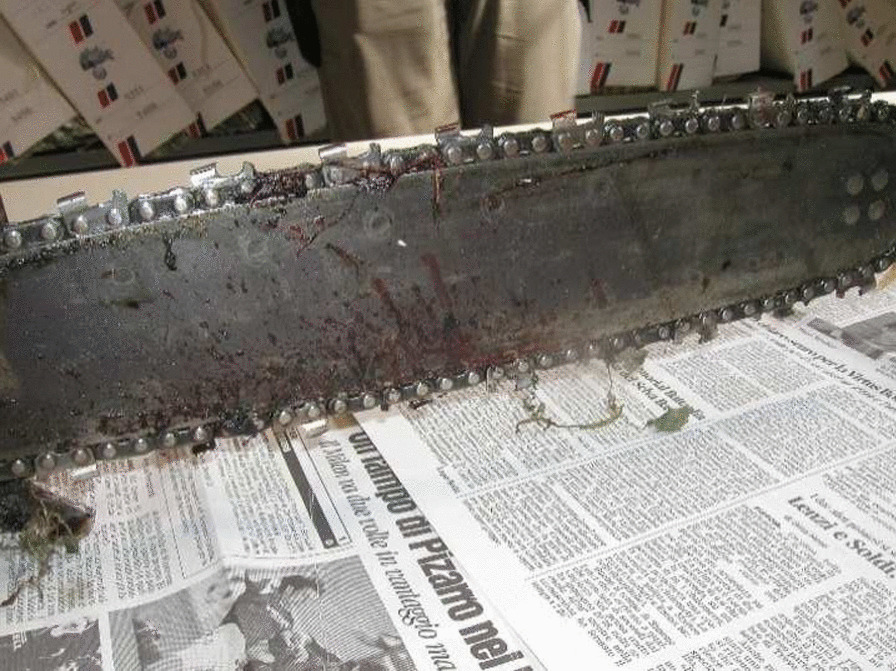
Detail of the blade

The chainsaw used in the present case is shown in Figs. 1, 2. It has a weight of 4.4 kg and a blade 41 cm in length. According to the instruction manual, the maximum number of revolutions is 2400 per minute without load. The saw runs only when the throttle of the hand switch is gripped, so when the grip is released, the blade of the saw stops. Only the victim’s fingerprints were found on the chainsaw. Examination of the wound (direction, orientation, depth) showed that it could only have been inflicted by the victim.

**Fig. 3:**
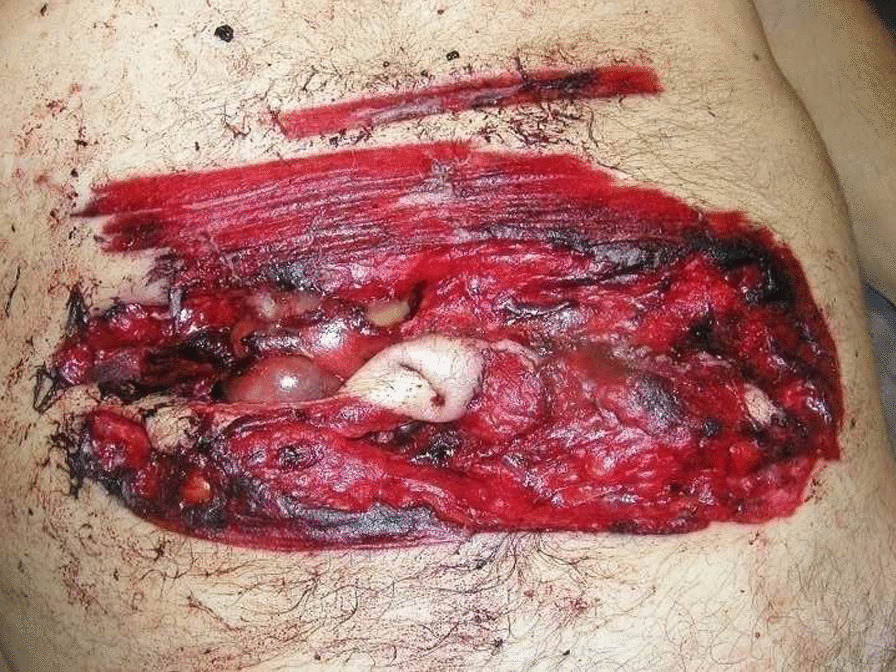
Abdominal incised wound surrounded by a wide reddish abrasion

Post-mortem examination was performed the day after the time of death. External examination showed a ligature mark around the neck, and a deep incised wound measuring 16 cm in width and up to 4.5 cm in length was encountered on the mesogastrium. The wound edges were generally torn, partly with grazed appearance (Fig. 3). The wound was surrounded by a wide reddish abrasion measuring 23 cm in width and 12 cm in length The skin mark on the neck was brownish yellow, and the anterior surface was parchment-like; furthermore, a narrow zone of reddened hyperemia at either margin of the mark was seen. Subconjunctival petechiae were observed. No other injuries, including hesitation injury, were noted externally.

**Fig. 4:**
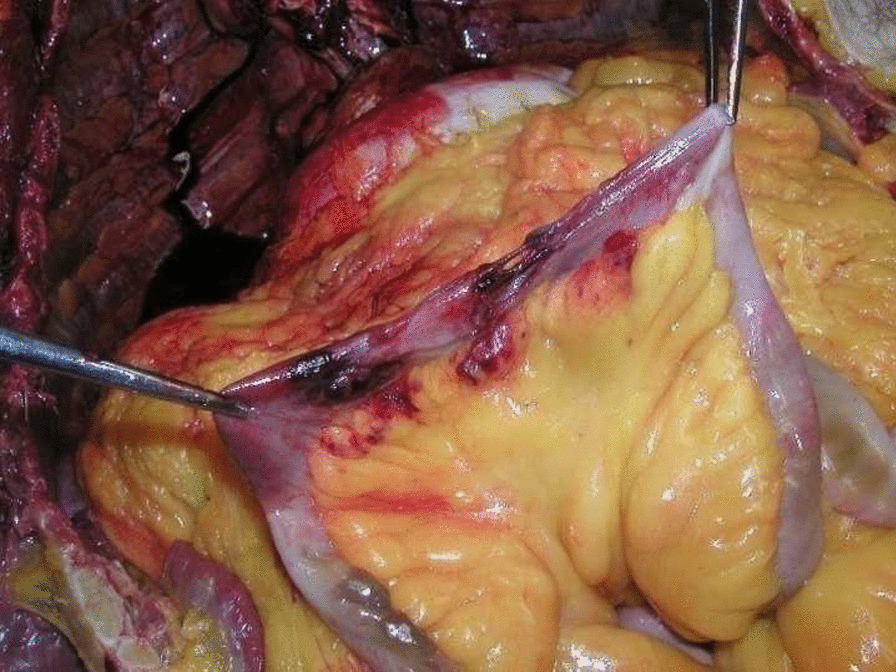
Lacerated intestinal loops

The autopsy showed hemorrhagic infiltration of the right omohyoid and sternocleidomastoid muscles, and the thyroid cartilage and the hyoid bone were intact. All the internal organs were intact and congested except for the intestinal loops below the chainsaw lesion, which were lacerated (Fig. 4), and 100 ml of blood was found in the abdominal cavity.

Histological examination confirmed the macroscopic findings and revealed hemorrhagic infiltration in the skin lesions and in the intestinal mucosa. Immunohistochemistry was also carried out, confirming the vitality of both injuries: the abdominal incised wound and the ligature mark. Body fluids were sampled for toxicological analysis: blood alcohol and drugs/illegal substances were negative.

The cause of death was deemed to be asphyxia due to hanging. The traumatic shock and exsanguination from the abdominal injury were not related to the fatal event.

## Discussion and conclusion

Fatalities attributed to power saws are exceptionally encountered in forensic medicine practice [1–13]. In our case, the wound was inflicted by a power saw in the abdominal area; a large amount of blood soaked the decedent’s clothes. A particular patterned injury was observed in other circumstances [14]. Since no one witnessed the death, from the medico-legal aspect, it is difficult to determine whether it is a case of homicide, suicide, or accident [13–17]. Typically, in suicide by self-cutting or stabbing, a clothing injury is absent, and perforation of the clothes covering the wound has been interpreted as an indication for homicide [18, 19]. This fact prompted suspicion of dealing with a case of homicide, because of the sweater lacerations, the multiple traumatic lesions of the corpse, and the involvement of various body parts.

Since the cadaver presented an apparently deep incised wound, establishing the manner and cause of death, excluding any third-party involvement, was crucial. Forensic investigations into such cases of unexplained death always require a wide differential diagnosis, including accidental suicide, instigation, or assistance to suicide and homicide.

The abdominal lesion appeared to be superficial: it involved the cutaneous–subcutaneous and muscular tissues with a superficial laceration of some intestinal loops. No vital organs were involved. The autopsy revealed that the abdominal lesion, even if responsible for abundant blood loss, was not a contributing cause of death, which was in fact determined to be asphyxia due to hanging.

Furthermore, the death scene investigation and the so-called psychological autopsy supported the mode of death as suicide and made it possible to corroborate the dynamics of the event: the man, who had been suffering from depression for many years, went to a familiar place (the garden beside the courtyard) and self-inflicted the abdominal injury with a chainsaw, a potentially life-threatening method. Failing that, he carried out the second phase, always choosing a familiar place, using a definitively deadly method, hanging himself.

The mental functioning of the depressed person is characterized by a cognitive triad. The cognitive triad is a cognitive-therapeutic view of the three key elements of a person’s belief system present in depression. The triad involves automatic, spontaneous, and seemingly uncontrollable negative thoughts about:

- The self (I’m worthless and ugly or I wish I was different);
- The world or environment (No one values me or people ignore me all the time);
- The future (I’m hopeless because things will never change or things can only get worse).

In addition to the cognitive triad, depressed people develop cognitive distortions, “automatic thoughts” not entirely under conscious control. The main cognitive distortions are:

- Arbitrary inference: drawing conclusions from insufficient or no evidence;
- Selective abstraction: drawing conclusions on the basis of just one of many elements of a situation;
- Overgeneralization: making sweeping conclusions based on a single event;
- Magnification: exaggerating the importance of an undesirable event;
- Minimization: underplaying the significance of a positive event;
- Personalization: attributing negative feelings of others to oneself.

Depressed people view their lives as devoid of pleasure or reward, presenting insuperable obstacles to achieving their important goals. This is often manifested as a lack of motivation and leads to the depressed person feeling further withdrawal and isolation. Decision paralysis results from the depressed patients’ pessimism and hopelessness.

They are pervaded by a strong death impulse that leads them first to suicide attempts and then to suicide, as an extreme expression of the desire to escape from problems that appear to be uncontrollable, interminable, and unbearable.

In the case presented, the man had attempted suicide 4 months earlier by ingesting some tablets of antidepressant drug (Seropram), for which he was hospitalized for 2 days. After 4 months, the man tried again to commit suicide.

There are numerous “proof” wounds that the man inflicted with the chainsaw in the abdominal area (Fig. 3) but all superficial. Unable to kill himself with the chainsaw, he decided to hang himself.

Therefore, this action is suggestive of an intense self-suppressive instinct that is always found in people committing such actions [20–22].

In fact, these subjects generally use multiple tools, with all the intrinsic damaging consequences.

In conclusion, we consider the case to be particularly interesting because it reiterates the importance of the “objective accuracy” that characterizes the medico-legal method in finding the correlation between injury and event.

Hence, a thorough post-mortem examination, in association with a precise study of the circumstantial data and medical history, and a detailed death scene investigation, led the authors to assume that they were dealing with a “complex suicide,” referring to a form of suicide in which more than one traumatic method is applied, simultaneously or consecutively.

In such cases, the autopsy would not have been sufficient to define and characterize the abdominal lesion. In this case, as well as in all the cases of suspected death, a psychological autopsy may have a key role, especially in analyzing the psychological profiles and determining the mental state of the decedent. There are many reasons why a psychological profile may be conducted, the most common one being to determine the cause or manners of death, whether it be by natural causes, suicide, homicide, or accident. A rather large amount of information must be collected to perform a psychological autopsy. Some of this information is personal (any history of drug/alcohol abuse, known stresses, lifestyle, relationships, and so on) [23] biographical information (birth date, occupation, marital or relationship status), any secondary information (criminal record, family history), and information gathered by interviewing family members of the deceased [21, 23, 24].

## Data Availability

The data and materials presented in the manuscript are preserved in the Department of Health Sciences, Section Forensic and Legal Medicine.

## References

[1] Segerberg-Konttinen M. Suicide by the use of a chain saw. J Forensic Sci. 1984;29:1249–52. 6502121

[2] Clark SP, Delahunt B, Thomson KG, Fernando TL. Suicide by band saw. Am J Forensic Med Pathol. 1989;10:332–4. 10.1097/00000433-198912000-00011 2589296

[3] Hartel V, Petkovits T, Brinkmann B. Unusual suicides with band saws. Arch Kriminol. 1989;184:168–74. 2629656

[4] Schiwy-Bochat KH. Verletzungsmuster bei suizidial tödlicher Kettensägenverletzung. Rechtsmedizin. 1992;2:71–3.

[5] Fernie CG, Gibson IH, Newton F. Chainsaw deaths: accident, suicide or murder? J Clin Forensic Med. 1994;1:109–12. 10.1016/1353-1131(94)90010-8 16371277

[6] Rainov NG, Burkert WL. An unusual suicide attempt using a circular saw. Int J Legal Med. 1994;106:223–4. 10.1007/BF01371342 8038117

[7] Betz P, Eisenmenger W. Unusual suicides with electric saws. Forensic Sci Int. 1995;75:173–9. 10.1016/0379-0738(95)01784-4 8586341

[8] Reul J, Bratzke H. Death caused by a chain saw—homicide, suicide or accident? A case report with a literature review (with 11 illustrations. Forensic Sci Int. 1999;105:45–9. 10.1016/s0379-0738(99)00096-1 10605075

[9] Campman SC, Springer FA, Henrikson DM. The chain saw: an uncommon means of committing suicide. J Forensic Sci. 2000;45:471–3. 10782976

[10] Judd O, Wyatt JP. Circular saw suicide. J Forensic Leg Med. 2007;14(4):235–7. 10.1016/j.jcfm.2006.06.030 17320459

[11] Tournel G, de Douit F, Balgairies A, Houssaye C, de Angeli B, *et al.* Unusual suicide with a chainsaw. J Forensic Sci. 2008;53(5):1174–7. 10.1111/j.1556-4029.2008.00795.x 18624895

[12] Gloulou F, Allouche M, Khelil B, Bekir O, Banasr A, *et al.* Unusual suicides with band saws: two case reports and a literature review. Forensic Sci Int. 2009;183:7–10. 10.1016/j.forsciint.2008.09.017 19019591

[13] Asano M, Nushida H, Nagasaki Y, Ueno Y. Suicide by a circular saw. Forensic Sci Int. 2008;182:e7–9. 10.1016/j.forsciint.2008.09.006 18980819

[14] Focardi M, Angelino A, Defraia B, Franchi E, Pinchi V. Fatal entrapment in a pool drainage system: a case report. Forensic Sci Med Pathol. 2019;15(4):638–41. 10.1007/s12024-019-00127-0 31183615

[15] Focardi M, Gualco B, Norelli GA. Accidental death in autoerotic manouvres. Am J Forensic Med and Path. 2008;1:64–8. 10.1097/PAF.0b013e3181651b79 19749620

[16] Karakasi M, Pavlidis P, Vasilikos E, Anestakis D, Raikos N. Complex suicide involving pyrethroid ingestion (mosquito coils) and fatal self-wounding by sharp force. Soud Lek. 2017;62(4):45–50. 29227118

[17] Focardi M, Bugelli V, Defraia B, Gualco B, Norelli GA. Accidental death in autoerotic maneuvers: case series. Rom J Leg Med. 2018;26:258–63.

[18] Ohsima T, Kondo T. Eight cases of suicide by self-cutting or -stabbing: consideration from medico-legal viewpoints of differentiation between suicide and homicide. J Clin Forensic Med. 1997;4:127–32. 10.1016/s1353-1131(97)90092-4 15335572

[19] Karlsson T. Multivariate analysis (‘Forensiometrics’)—a new tool in forensic medicine. Differentiation between sharp force homicide and suicide. Forensic Sci Int. 1998;94:183–200. 10.1016/s0379-0738(98)00065-6 9717272

[20] Bugelli V, Gherardi M, Focardi M, Pinchi V, Vanin S, *et al.* Decomposition pattern and insect colonization in two cases of suicide by hanging. Forensic Sci Res. 2018;3(1):94–102. 10.1080/20961790.2017.1418622 PMC6197082 30483656

[21] Arafat SMY. Psychological autopsy study in Bangladesh: an unmet need to formulate preventive strategy of suicide. Asian J Psychiatr. 2019;43:85–6. 10.1016/j.ajp.2019.05.019 31102914

[22] Zhang J, Liu X, Fang L. Combined effects of depression and anxiety on suicide: a case–control psychological autopsy study in rural China. Psychiatr Res. 2019;271:370–3. 10.1016/j.psychres.2018.11.010 PMC6382523 30529321

[23] Gatti U, Fossa G, Gualco B, Caccavale F, Ceretti A, Ciliberti R, *et al.* Italy. In: Junger-Tas J, Marshall I, Enzamann D, Killias M, Steketee M, Gruszczynska B, editors., *et al.*, Juvenile delinquency in Europe and beyond: results of the second international self-report delinquency study. New York: Springer; 2010. p. 227–44.

[24] Focardi M, Pinchi V, Defraia B, Gualco B, Varvara G, Norelli GA. Newborn screening of inherited metabolic disorders: the Italian situation. J Biol Regul Homeost Agents. 2016;30(3):909–14. 27655520

